# The Airplane Cabin Microbiome

**DOI:** 10.1007/s00248-018-1191-3

**Published:** 2018-06-06

**Authors:** Howard Weiss, Vicki Stover Hertzberg, Chris Dupont, Josh L. Espinoza, Shawn Levy, Karen Nelson, Sharon Norris, The FlyHealthy Research Team

**Affiliations:** 1 School of Mathematics, The Georgia Institute of Technology, 686 Cherry St. NW, Atlanta, GA 30313 USA; 2 Nell Hodgson Woodruff School of Nursing, Emory University, 1520 Clifton Rd. NE, Atlanta, GA 30322 USA; 3 J. Craig Venter Institute, 4120 Capricorn Lane, La Jolla, CA 92037 USA; 4 HudsonAlpha Institute for Biotechnology, 601 Genome Way, Huntsville, AL 35806 USA; 5 J. Craig Venter Institute, 9714 Medical Center Drive, Rockville, MD 20850 USA; 6 Boeing Health Services, The Boeing Company, 3156 160th Ave. NE, Bellevue, WA 98008-2245 USA

**Keywords:** Commercial airplanes, Microbiome, Bacteria, Pandemic, Respiratory infection

## Abstract

Serving over three billion passengers annually, air travel serves as a conduit for infectious disease spread, including emerging infections and pandemics. Over two dozen cases of in-flight transmissions have been documented. To understand these risks, a characterization of the airplane cabin microbiome is necessary. Our study team collected 229 environmental samples on ten transcontinental US flights with subsequent 16S rRNA sequencing. We found that bacterial communities were largely derived from human skin and oral commensals, as well as environmental generalist bacteria. We identified clear signatures for air versus touch surface microbiome, but not for individual types of touch surfaces. We also found large flight-to-flight beta diversity variations with no distinguishing signatures of individual flights, rather a high between-flight diversity for all touch surfaces and particularly for air samples. There was no systematic pattern of microbial community change from pre- to post-flight. Our findings are similar to those of other recent studies of the microbiome of built environments. In summary, the airplane cabin microbiome has immense airplane to airplane variability. The vast majority of airplane-associated microbes are human commensals or non-pathogenic, and the results provide a baseline for non-crisis-level airplane microbiome conditions.

**Electronic supplementary material:**

The online version of this article (10.1007/s00248-018-1191-3) contains supplementary material, which is available to authorized users.

## Introduction

With over three billion airline passengers annually, the risk of in-flight transmission of infectious disease is a vital global health concern [1, 2]. Over two dozen cases of in-flight transmission have been documented, including influenza [3–7], measles [8, 9], meningococcal infections [10], norovirus [11], SARS [12, 13], shigellosis [14], cholera [15], and multi-drug resistant tuberculosis [1, 16–18]. Studies of SARS [12, 13] and pandemic influenza (H1N1p) [19] transmission on airplanes indicate that air travel can serve as a conduit for the rapid spread of newly emerging infections and pandemics. Further, some of these studies suggest that the movements of passengers and crew (and their close contacts) may be an important factor in disease transmission. In 2014, a passenger infected with Ebola flew on Frontier airlines the night before being admitted to a hospital [20]. Luckily, she did not infect anybody during that trip.

Despite many sensational media stories and anecdotes, e.g., “Flying The Filthy Skies” [21] or “The Gross Truth About Germs and Airplanes” [22], the true risks of in-flight transmission are unknown. An essential component of risk assessment and public health guidance is characterizing the background microbial communities present, in particular those in the air and on common touch surfaces. Next-generation sequencing has the potential to identify all bacteria present via their genomes, commonly called the microbiome. There have been a few previous studies of the bacterial community in cabin air [23–26], but none, to our knowledge, on airplane touch surfaces. These studies estimated total bacterial burden of culturable cells present, and applied early forms of 16S rRNA sequencing and bioinformatics, claiming species-level resolution. At the time of these studies, there were far fewer reference genomes with which to align. Although these were at the vanguard of research of the microbiome of built environments, 10 years later, current methods and protocols are significantly more rigorous.

The microbiome of the built environment is an active research area. Using a wide range of methods, authors have studied the microbiomes of classrooms [27–29], homes [30–32], offices [33, 34], hospitals [35], museums [36], nursing homes [37], stores [38], and subways [39–41]. Several of these studies, particularly those of classrooms and offices, identified significant quantities of *Lactobacillus* on seats. With the exception of the hospital microbiome, all of these studies indicate that the main microbiome constituents, at the family level, are human commensal and environmental bacteria. What else could they be?

Airplane environments are unique to the examples listed above. Special features include very dry air, periodic high occupant densities, exposure to the microbiota of the high atmosphere, and long periods during which occupants have extremely limited mobility. Thus, one might expect that the airplane cabin microbiome might differ considerably from those of other built environments. Another key difference is that in an airplane cabin, it is difficult to avoid a mobile sick person, or one sitting in close proximity.

In another publication [42], we describe behaviors and close contacts of all passengers and flight attendants in the economy cabin on ten flights of duration 4 hours or more, the FlyHealthy™ Study. FlyHealthy™ has provided first detailed understanding of infectious disease transmission opportunities in an airplane cabin. In addition to quantifying the opportunities, we wanted to understand the infectious agents present in an airplane cabin that might be transmitted during these opportunities.

To this end, we identified the microbiota present on these flights, allowing characterization of the airplane cabin microbiome. We hypothesized that the airplane cabin microbiome differs from that of other built environments due to the above-stated reasons. Since the majority of flights were during the seasonal flu epidemic in either the originating city or the destination city, we were interested to determine if we could detect influenza virus in our samples. Since the transmission opportunities we characterized in the first part of the FlyHealthy™ study were those that would allow transmission by large droplets, we were interested in sampling air as well as touch surfaces (fomites). Key questions related to differences between types of samples (air versus touch surfaces), pre- to post-flight changes, and changes from flight-to-flight in the “core” airplane cabin microbiome.

## Results

### Airplane Cabin Bacterial Communities in the Air and on Touch Surfaces

**Fig. 1:**
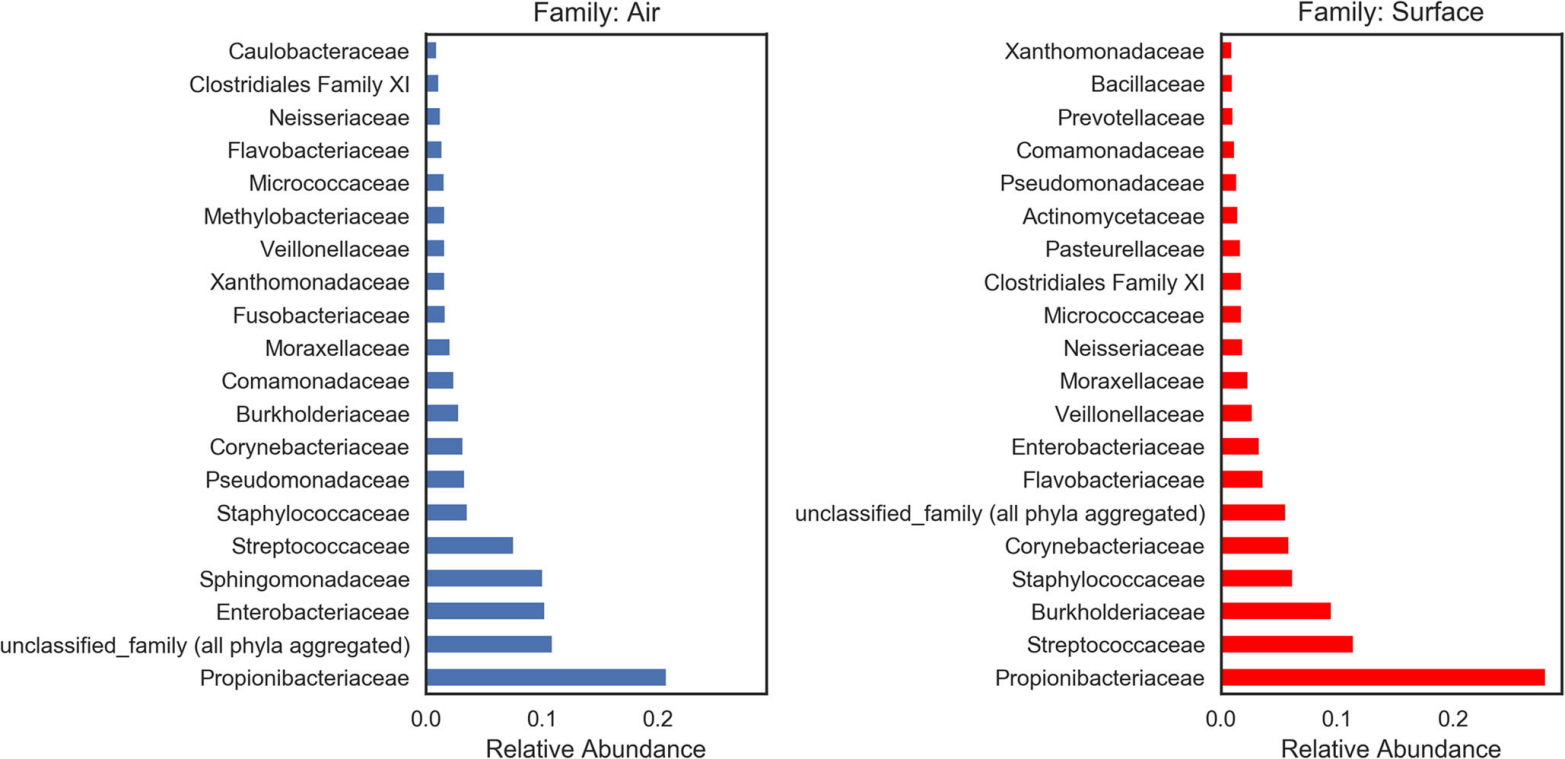
Most prevalent families in air (left) and touch surface samples (right) by relative abundance (proportion of families)

Skin commensals in the family *Propionibacteriaceae* dominate both air (~ 20% post-filtered reads) and touch surfaces (~ 27% post-filtered reads). There is substantial overlap of the top 20 families in air and touch surface samples (Fig. 1). The top ten families in both air and fomites additionally contain *Enterobacteriaceae*, *Staphylococcaceae*, *Streptococcaceae*, *Corynebacteriaceae*, and *Burkholderiaceae*. The environmental bacteria *Sphingomonadaceae* is quite prevalent in the air, but much less so on touch surfaces. Note that “unclassified family” aggregates different families from different higher level taxa. The top OTUs are shown in SM Fig. 1.

OTUs within the genera *Propionibacterium* and *Burkholderia* were present in every sample and two OTUs, annotated as genus *Staphylococcus* and *Streptococcus* (*oralis*), were present in all but one sample. These four OTUs are contained in three phyla: *Actinobacteria*, *Proteobacteria*, and *Firmicutes*, and comprise the “core” airplane cabin microbiome.

### Air and Touch Surface Communities Have Discernible Signatures, but There Are No Discernible Signatures of Touch Surface Types

**Fig. 2:**
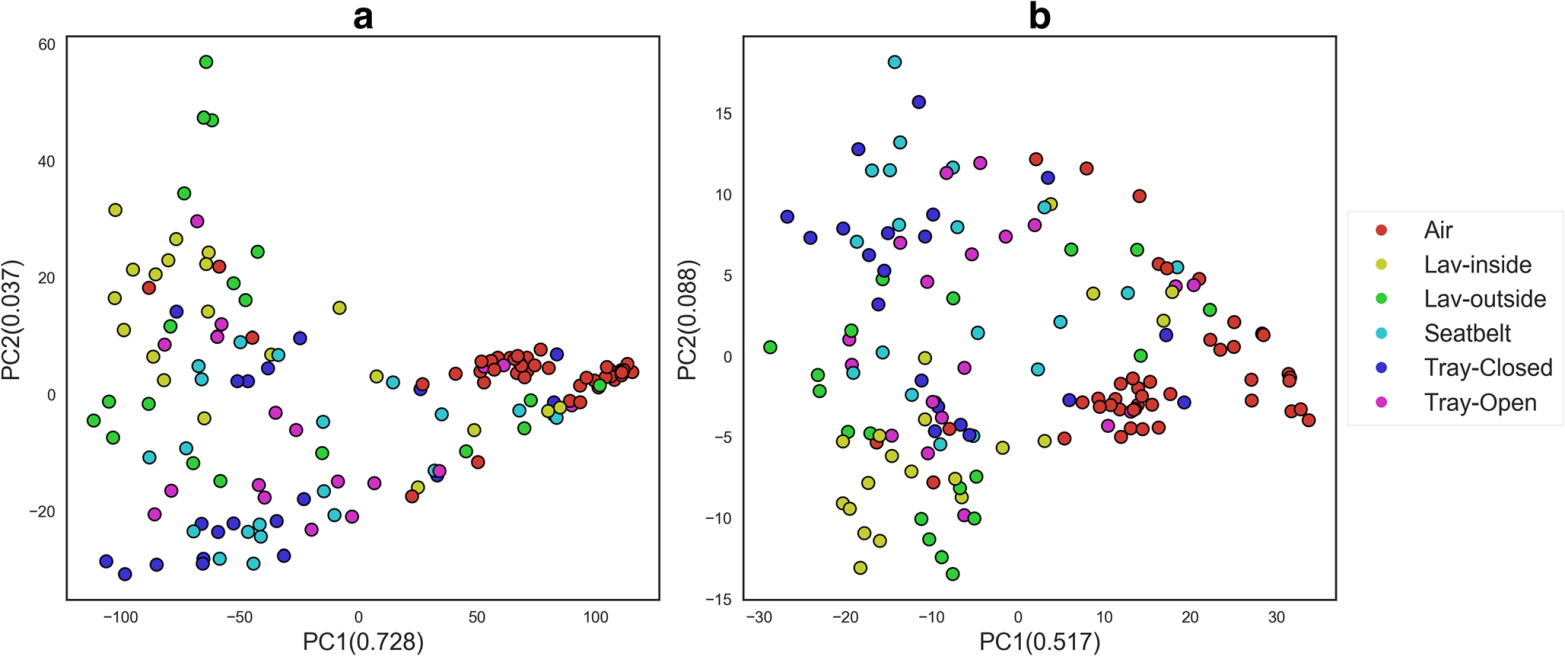
Scatterplot of the logs of the first two principal components, colored by sample source. **a** Families. **b** OTUs

Figure 2 shows the results of the principal component analysis (PCA) on a log-scale of families of all samples over all ten flights. The associated scree plot (SM Fig. 3) indicates that the vast majority (73%) of the variability is captured by first principal component, about an order of magnitude more than that captured by PC2. We observe that the air samples are primarily positive on PC1 and, in fact, greater than 50, while the touch surface samples are largely negative. When combined with the variance explained by PC1 (Fig. 2b), this indicates a clear signature of the air community. The complement is the signature of the touch surface community. There is a potpourri of touch surface types in the figure, again indicating the lack of clear signature of individual touch surface type. There are no statistically significant differences of alpha diversity between air and fomites as measured by any of six indices (SM Fig. 2).

**Fig. 3:**
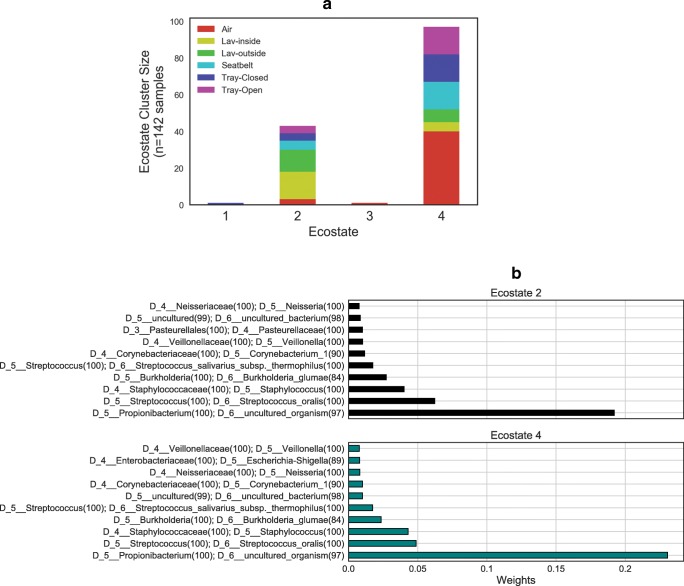
Results of iDMM analysis indicating two distinct ecostates. **a** Composition of the four ecostates identified in the iDMM analysis. **b** Most prevalent OTUs identified in the two ecostates associated with cabin air

Use of an infinite Dirichlet–multinomial mixture **(**iDMM) model [43] identified four clusters (or ecostates), with ecostate 4 containing the vast majority of air samples, though it also includes many fomite samples as well (Fig. 3a). Figure 3b shows the diagnostic OTUs present in this air cluster and their weights. Note that the weights are an essential component of this characterization.

**Fig. 4:**
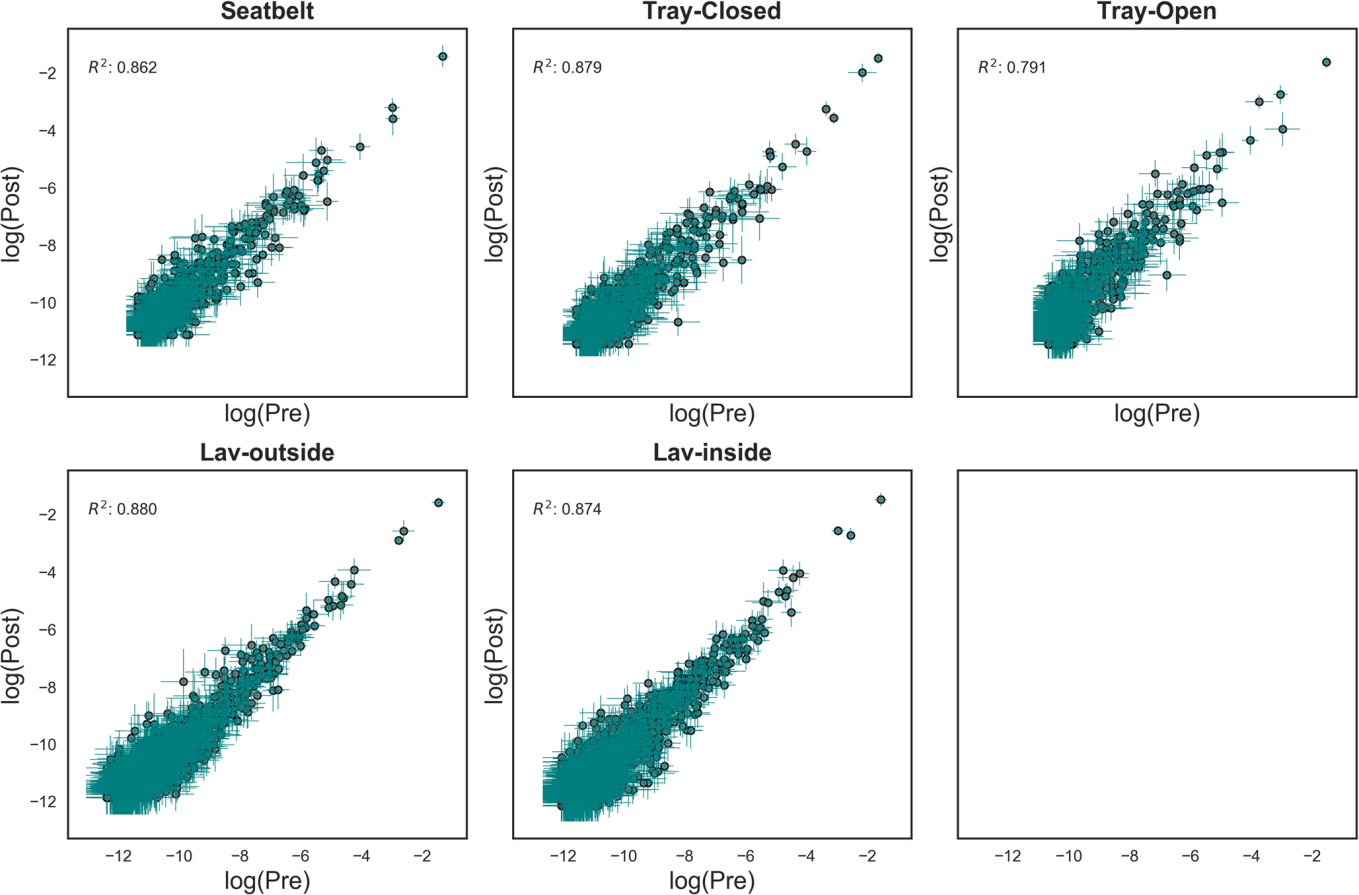
Logged average number of reads for OTUs from pre- to post-flight for each touch surface (fomite) type

Another important question is whether bacterial communities change discernibly during flight? Again, Fig. 4 shows the admixture of pre- and post-flight communities in the touch surface samples. Note the linearity of these scatterplots of the logged average number of reads for OTUs from pre- to post-flight for each touch surface type. There is no discernible pattern of change of pre-flight to post-flight communities.

**Fig. 5:**
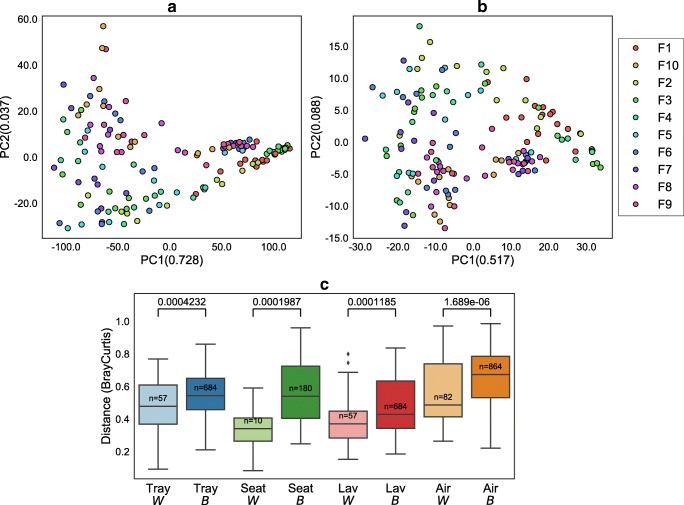
Beta diversity of samples. Scatterplot of the first two principal components of the beta diversity analysis, for **a** OTU-level and **b** family-level abundance, based on a Bray-Curtis distance. **c** Distributions of Bray-Curtis distances for different touch surface types, within and between flights

A final key question is whether bacterial communities in the cabin air change discernibly from flight to flight? For example, is there a difference between east-bound versus west-bound flights? A principle component analysis at both the family and OTU levels shows a wide variation with no clustering by flight (Fig. 5). Furthermore, without exception, between-flight (B) beta diversity is statistically higher than within-flight (W) beta diversity, that is, each flight is already starting with microbiomes that are likely different from other flights.

## Discussion

Toward the goal of characterizing the airplane cabin microbiome, our study team flew on ten transcontinental US flights on which we collected 229 air and touch surface samples. We employed highly stringent quality control criteria during sampling, sample extraction, 16S rRNA gene sequencing, and the bioinformatics pipeline. The observed microbial communities, when merged across samples, are comprised of human commensals and common environmental (water and soil) genera. We identified a “core” airplane cabin microbiome containing OTUs within the genera *Propionibacterium, Burkholderia* (*glumae*), *Staphylococcus*, and *Streptococcus* (*oralis*). We identified clear OTU signatures for the air microbiome, but not for individual touch surface types. We found no meaningful differences between air and touch surfaces with respect to alpha diversity measures. Finally, we found no systematic pattern of change from pre- to post-flight.

We also found large flight-to-flight variations with no distinguishing signatures of individual flights. This would suggest that each flight starts with a different microbiome from other flights, which would greatly hinder pre-and post-flight microbiome comparisons (e.g., Fig. 4) that aggregate samples between flights. A methodological implication is that aggregating communities between flights for statistical analyses is problematic. Instead, sample replication must be derived from within a flight in order to determine how passengers alter the airplane cabin microbiome. Every plane being different in terms of its microbiome suggests that each retains aspects of its historical living microbiome, that is, the passengers. The development of a cleaning routine that erases much of this inherited microbiome could be a powerful preventative measure against the spread of disease.

*Propionibacterium* is a genus of the phylum *Actinobacteria*, comprised of commensal bacteria that live on human skin and commonly implicated in acne. *Burkholderia glumae* is a species of the phylum *Proteobacteria* and is a soil bacterium. *Staphylococcus* is a genus of the phylum *Firmicutes* that is found on the skin and mucus membranes of humans. Most species of *Staphylococcus* are harmless. *Streptococcus oralis*, a species of the phylum *Firmicutes*, is normally found in the oral cavities of humans. These constituents of the core airplane cabin microbiome are usually harmless to humans unless an unusual opportunity for infection is present, such as a weakened immune system, an altered gut microbiome, or a breach in the integumentary system.

While airplane cabins are certainly examples of built environments, there are unique features. These include very dry air, periodic high occupant densities, exposure to the microbiota of the high atmosphere, long periods during which occupants have extremely limited mobility, and it is difficult to avoid a mobile sick person or one sitting in close proximity. Half of the cabin air is recycled after passing through a bank of HEPA filters, and the other half is taken from the outside. Furthermore, the airline’s cabin cleaning policy is to disinfect all hard surfaces whenever the plane “overnights,” and all touch surface samples were taken from hard surfaces. Different airlines have different cabin disinfection protocols and supervise their cabin cleaning staff in different ways.

Despite the uniqueness of the airplane cabin as a built environment, our findings are surprisingly consistent with other recent studies of the microbiome of built environments. This consistency is reassuring in light of frequent sensationalistic media stories about dangerous germs found on airplanes. For this reason, there is no more risk from 4 to 5 hours spent in an airplane cabin than 4–5 hours spent in an office, all other exposures being the same. Our microbiome characterization also provides a baseline for non-crisis level airplane microbiome conditions.

It is not possible to make quantitative comparisons to other studies which used different primers and different sequencing methods and technologies. For example, the genus *Propionibacterium* is a core component of the airplane cabin microbiome, but by choice of primers, the most common species, *Propionibacterium acnes*, a common skin commensal, was excluded from discovery in the New York City subway microbiome study*.*

Although different primers and sequencing techniques were used, the core microbiome identified in the Boston subway system study has significant overlap with airplane cabins [41]. *Corynebacteriaceae*, a skin commensal, appeared in nearly every subway sample, and while we do not include it in the airplane cabin core list, it was present in all but ten of our samples. A study of the microbiome of the International Space Station, the only other airborne built environment that has been studied, led to the same conclusion [44], as did two studies of office spaces [33, 34].

A number of previous studies identified large amounts of *Lactobacillus*, but *Lactobacillaceae* did not appear in our list of 20 most prevalent families in our touch surface samples. *Lactobacillus* is commonly found in vaginal microbiota, suggesting that it should be found on surfaces where women sit. Many other studies of the built environment have sampled seats, and thus, it is not surprising to find *Lactobacilli* present in those environments. We did not sample from the seat fabric where passengers sat; thus, the absence of *Lactobacilli* in the 20 most prevalent families is to be expected.

Airplanes fly through clouds. The narrow-body twin-engine models on which we flew use about 50% bleed (outside) air to refresh the cabin air throughout the flight. A study of the microbiome of clouds finds some members of the *Propionibacterium* and *Burkholderia* families in their core, as well as *Streptococcus* in some samples [45]. A more recent study of cloud water found *Burkholderia, Staphylococcus*, and *Streptococcus* in samples [46]. Interesting future research would be to ascertain the influence of the cloud microbiome on the airplane cabin microbiome.

In conclusion, our study found that although the microbiome of airplane cabins has large flight-to-flight variations, it resembles the microbiome of many other built environments. This work adds to the growing body of evidence characterizing the built environment. These investigations form critical linkages between the categories of environmental and human-associated microbial ecology, and thus must meet the challenges of both areas. Improvements in future studies should include incorporation of rich metadata, such as architectural and other design features, human-surface contacts, and environmental exposures, as well as determination of microbe viability and the mechanisms used to persist in the airplane cabin environment. Identification of microbes that can be transferred between passengers and specific fomites will be especially important in informing public health and transportation policy. We hope to undertake an analogous study on significantly longer, international flights, as well as at key locations at departing and arriving airports. An improved understanding of the airplane cabin microbiome and how it is affected by passengers and crew may lead ultimately to construction of airplane cabins that maintain human health.

## Materials and Methods

### Selection of Flights

Each of five round-trips, on non-stop flights, targeted a different west coast destination to provide data representative of transcontinental flights. We flew to San Diego, Los Angeles, San Francisco, and Portland, OR, between November 2012 and March 2013. We flew to Seattle, WA, in May 2013. We flew on narrow-body twin-engine aircraft, with all but one flight on a specific model. Our movement data are representative of passenger and crew movements in a single aisle “3 + 3” economy cabin configuration.

### Air Sampling Methods

The two air sampling pumps used were model SKC AirChek XR5000. These were located in a seat at the back of the economy class cabin. Both pumps sampled at 3.5 liters per second, the NIOSH protocol for stationary sampling and approximately the normal breathing rate of adults.

Just prior to each sampling, each pump was calibrated using a MesaLab Defender Calibrator. Air samples of 30-min duration were collected onboard the aircraft during five distinct sampling intervals. Once the pilot announced the flight time, we calculated the quarter-way point, halfway point, and three quart-way point. Thus, the five sampling periods were pre-boarding and boarding, Q1 ± 15 min, Q2 ± 15 min, Q3 ± 15 min, and touchdown to end of deplaning. In addition, one sample was collected throughout the whole flight from 10,000 ft on ascent to 10,000 ft on descent. Flight 2 only has data for four time points. Following each sampling period, the sampling cartridges were wrapped with Teflon tape, labeled, logged, and placed in a cooler with chemical ice packs.

### Fomite Sampling Methods

Prior to each flight, we prepared an ordered list of seven randomly selected seats, of which the first two occupied seats, as confirmed by the gate agent prior to boarding, were sampled. We also randomly chose a rear lavatory door (port or starboard) for sampling.

We swabbed the laboratory door handles using Bode SecurSwab DNA Collector dual swabs, placing three drops of DNA- and RNA-free water on one of the two swabs, then, swabbing in one direction within a 9 cm × 9 cm template, and finally swabbing in the perpendicular direction within the same template. Afterwards, we placed each swab into its secure tube, labeled it, logged it, and placed it into a cooler on a chemical ice pack.

We sampled three touch surfaces at each passenger seat—the inside tray table, the outside tray table, and the seat belt buckle. Using the templates and the dual swabs, we sampled the bottom corners of each side of the tray table as described above. We did not use the template to swab the seat belt buckle; rather, we swabbed the entire upper surface in one direction and then in the perpendicular direction. We placed each swab into its secure tube, labeled it, logged it, and placed it into a cooler on a chemical ice pack.

Material from the two swabs was combined in Tris Buffer and homogenized per kit instructions. The air filters were similarly prepared. DNA isolations were performed using the Power Soil kit (MoBio Laboratories, Carlsbad, CA) according to the manufacturer’s directions with an elution volume of 50 μl. The 16S rRNA gene was amplified for sequencing using the 515F primer (5′ GTGCCAGCMGCCGCGGTAA 3′) and 806R primer (5′ GGACTACHVGGGTWTCTAAT 3′) [47]. The 16S rRNA gene-specific primers were tailed with Illumina adaptor sequences to allow a secondary PCR to add indexing barcodes and full Illumina adaptor sequences to support paired-end sequencing. Libraries were pooled for sequencing in batch sizes of 48 samples per batch and sequenced on the Illumina MiSeq at HudsonAlpha Biosciences. Paired-end sequencing with a read length of 150 bases per read was used, providing a small overlap at the end of each read to facilitate assembly of the paired-end sequencing reads to a single fragment of ~ 290 bp representing the V4 region of the 16S rRNA gene. In reality, the reverse read was of very low quality preventing assembly for forward and reverse reads. Therefore, only quality trimmed forward reads were used for all downstream analyses. The 16S sequence data have been deposited in the National Center for Biotechnology Information (NCBI) database on BioProject accession number: PRJNA420089 and at the Sequence Read Archive (SRA) under Accession IDs SRR6330835–SRR6330871.

Reads were de-multiplexed according to the barcodes and trimmed of barcodes and adapters. Following the initial processing of the sequence data, sequences were combined, dereplicated, and aligned in mothur (version 1.36.1) [48] using the SILVA template (SSURef_NR99_123) [49]; subsequently, sequences were organized into clusters of representative sequences based on taxonomy called operational taxonomic units (OTU) using the UPARSE pipeline [50]. Initial filtering of the samples ensured discarding OTUs containing less than five sequences. Libraries were normalized using metagenomeSeq’s cumulative sum scaling method [51] to account for library size acting as a confounding factor for the beta diversity analysis. Moreover, in addition to discarding singletons, OTUs that were observed fewer than seven times in the count data were also filtered out to avoid the inflation of any contaminants that might skew the diversity estimates.

## Electronic supplementary material

ESM 1 (DOC 4945 kb)
