# Supplementary material for: The Airplane Cabin Microbiome
Source: Microb Ecol. 2018 Jun 6;77(1):87–95. doi: 10.1007/s00248-018-1191-3 (PMC6318343; doi:10.1007/s00248-018-1191-3)

Supplemental Figures

**Figure SM1.** Alpha diversity indices of samples by sample type (air or surface).

**Figure SM2.** The 20 most prevalent OTUs in all (top), air (middle), and surface (bottom) samples.

**Figure SM3:** Scree plots for principal components analysis. A) Blow-up of cumulative variance accounted for by the first 10 principal components at the family level; B) Blow-up of cumulative variance accounted for by the first 10 principal components at the OTU level; C) Cumulative variance accounted for by all principal components at the family level; D) Cumulative variance accounted for by all principal components at the family level.


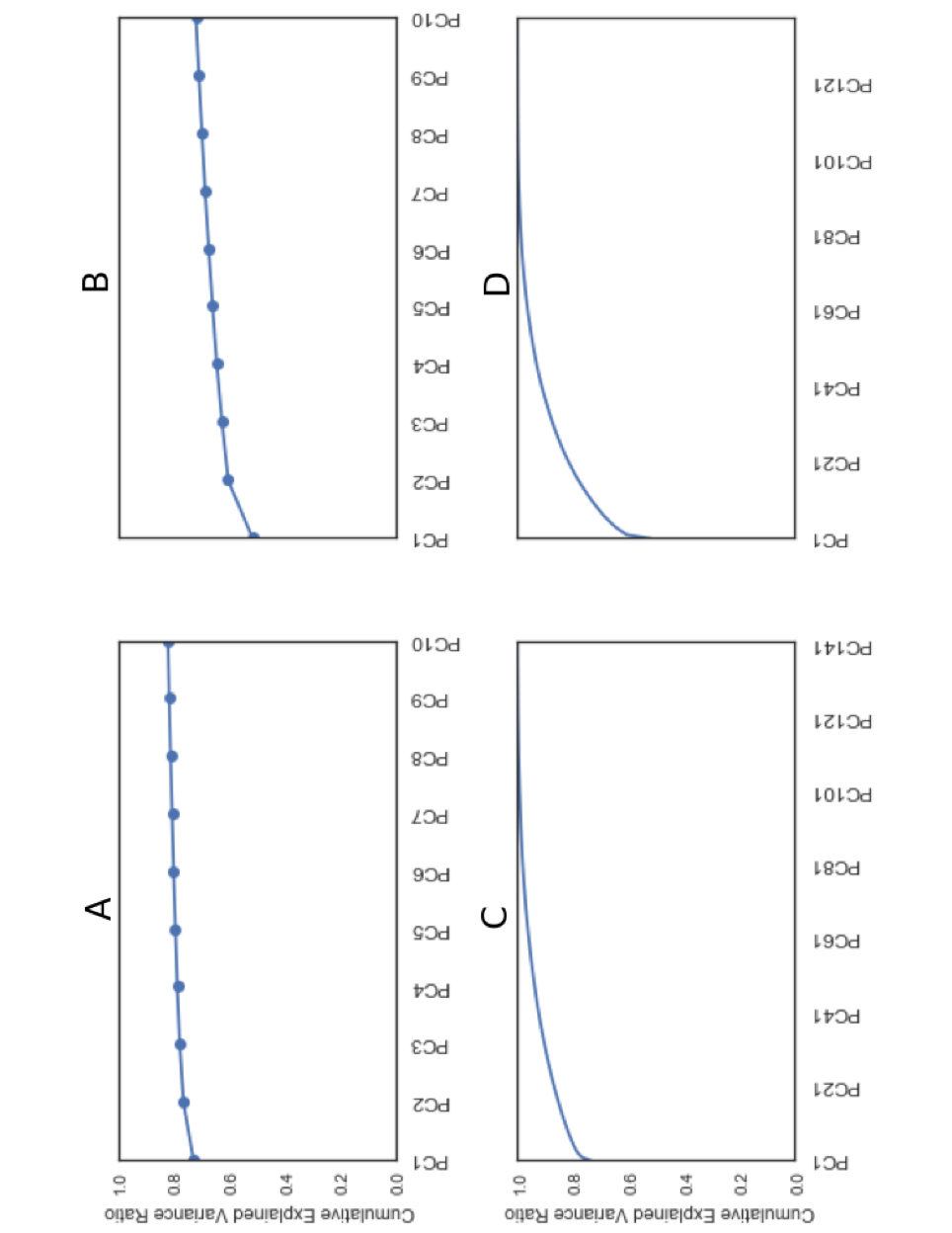

Supplement: Supplementary file 1 — (DOC 4945 kb) [file 248_2018_1191_MOESM1_ESM.doc]
